# PBK/TOPK: A Therapeutic Target Worthy of Attention

**DOI:** 10.3390/cells10020371

**Published:** 2021-02-11

**Authors:** Ziping Han, Lingzhi Li, Yuyou Huang, Haiping Zhao, Yumin Luo

**Affiliations:** 1 Institute of Cerebrovascular Disease Research and Department of Neurology, Xuanwu Hospital of Capital Medical University, Beijing 100053, China; hanzip@xwhosp.org (Z.H.); lingzhi08@ccmu.edu.cn (L.L.); youzaimedicu@126.com (Y.H.); 2 Beijing Geriatric Medical Research Center and Beijing Key Laboratory of Translational Medicine for Cerebrovascular Diseases, Beijing 100053, China; 3 Beijing Institute for Brain Disorders, Beijing 100053, China

**Keywords:** PBK/TOPK, mitosis, malignancy, ischemia, inhibitor

## Abstract

Accumulating evidence supports the role of PDZ-binding kinase (PBK)/T-lymphokine-activated killer-cell-originated protein kinase (TOPK) in mitosis and cell-cycle progression of mitotically active cells, especially proliferative malignant cells. PBK/TOPK was confirmed to be associated with the development, progression, and metastasis of malignancies. Therefore, it is a potential therapeutic target in cancer therapy. Many studies have been conducted to explore the clinical applicability of potent PBK/TOPK inhibitors. However, PBK/TOPK has also been shown to be overexpressed in normal proliferative cells, including sperm and neural precursor cells in the subventricular zone of the adult brain, as well as under pathological conditions, such as ischemic tissues, including the heart, brain, and kidney, and plays important roles in their physiological functions, including proliferation and self-renewal. Thus, more research is warranted to further our understanding of PBK/TOPK inhibitors before we can consider their applicability in clinical practice. In this study, we first review the findings, general features, and signaling mechanisms involved in the regulation of mitosis and cell cycle. We then review the functions of PBK/TOPK in pathological conditions, including tumors and ischemic conditions in the heart, brain, and kidney. Finally, we summarize the advances in potent and selective inhibitors and describe the potential use of PBK/TOPK inhibitors in clinical settings.

## 1. Introduction

T-lymphokine-activated killer-cell-originated protein kinase (TOPK), also known as PDZ-binding kinase (PBK), is a novel mitotic serine/threonine protein kinase [1,2]. PBK/TOPK is overexpressed in various actively proliferative cells, including malignant tumor cells, as well as normal cells, such as sperm cells. The transcription, activation, and degradation of PBK/TOPK is regulated by mitosis progression, which is mediated by a series of proteins. As a mitotic kinase, PBK/TOPK is important for the proliferation, progression, and metastasis of many cancers, including leukemia and myeloma, among others [3,4]. Upregulation of PBK/TOPK has also been proven to be associated with cancer diagnosis and prognosis, and, thus, it may be a potential therapeutic target in various malignant tumors. Thus, various potent PBK/TOPK inhibitors have been developed. The function of PBK/TOPK as an emerging target for cancer-specific therapeutics has been reviewed previously by Herbert et al. [5]. Furthermore, ours and other research teams also demonstrated that PBK/TOPK plays crucial roles in ischemic injury and is involved in protection against ischemia and ischemic postconditioning [6,7,8]. This protective effect of PBK/TOPK in the context of ischemia challenged the development of PBK/TOPK inhibitors in antitumor therapy, and more research is required to further explore its role and underlying mechanisms, to translate its applicability to clinical studies.

## 2. Identification of PBK/TOPK

The human homologue of the Drosophila tumor suppressor Discs-large (hDlg) is a membrane-associated guanylate kinase homologue (MAGUK) [9]. Several proteins that bind to hDlg have been demonstrated to be involved in cell growth control, including in the pathogenesis of adenomatous polyposis coli [10,11,12,13]. In 2000, Gaudet et al. sought to elucidate the signal transduction pathway through which hDlg regulates cellular proliferation, and they first identified a 322 amino acid serine/threonine kinase, using a two-hybrid screen and named it PDZ-binding kinase (PBK) [2]. At the same time, Abe et al. cloned a novel protein kinase from a lymphokine-activated killer T (T-LAK) cell subtraction cDNA fragment library and named it T-LAK cell-originated protein kinase (TOPK) [1]. This gene was later confirmed to be similar to that found by Gaudet et al., and this novel gene, or its protein product, was called PBK/TOPK.

## 3. General Features of PBK/TOPK

Unlike hDlg, which is ubiquitously expressed, the expression and distribution of PBK/TOPK varies. It is abundant in highly proliferative tissues, such as the placenta, testis, T-LAK cells, activated lymphoid cells and lymphoid tumors, but it is expressed at a very low level or is even absent in non-proliferative normal tissues, such as normal adult brain tissue (with the exception of the subependymal zone and early postnatal cerebellar external layer, which is enriched with rapidly proliferating progenitor cells) [14,15]. Regarding its intracellular distribution, PBK/TOPK is expressed both in the cytoplasm and the nucleus; however, it may be expressed exclusively in the nucleus of tumor cells or in mitosis, particularly around chromosomal surfaces during prophase and metaphase [16,17]. Moreover, both PBK/TOPK protein expression and its activation are subject to cell-cycle regulation. Using the thymidine double-block method, PBK/TOPK protein expression was demonstrated to increase and peak at 8 h after being released from the cell-cycle block during G2 to M phase in HeLa cells, and this was accompanied by the upregulation of its phosphorylation activity [18]. 

**Figure 1:**
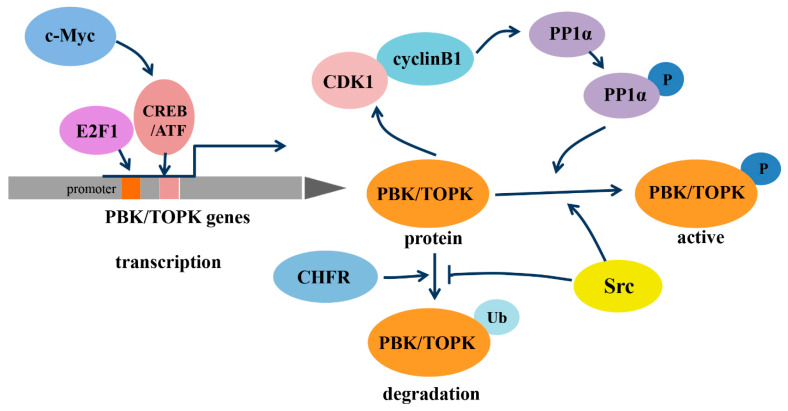
Regulation of transcription, phosphorylation and degradation of PDZ-binding kinase/T-lymphokine-activated killer-cell-originated protein kinase (PBK/TOPK). Abbreviations: c-Myc, the human cellular homologue of the v-myc oncogene of avian myelocytomatosis retrovirus MC29; E2F1, transcription factor E2F contained eight E2F genes, including E2F1-8; CREB/ATF, cyclic AMP-responsive element binding protein/activating transcription factor; CDK1, cyclin-dependent kinase 1; PP1α, protein phosphatase 1 alpha; CHFR, checkpoint protein with FHA and RING domains; Src, a non-receptor tyrosine kinase.

As a member of the MEK3/6-related MAPKK family, PBK/TOPK shares the characteristic serine/threonine protein kinase subdomains and a C-terminal PDZ-binding T/SXV motif, which enables it to bind specifically to the PDZ2 domain of hDlg or other PDZ-containing proteins. Activated PBK/TOPK is also able to phosphorylate P38 MAPK and histone H3, among other proteins [2,19,20]. However, the phosphotransferase activity of PBK/TOPK appears to be regulated in a cell-cycle-dependent manner; that is, it is activated following its phosphorylation by cyclin-dependent kinase 1 (CDK1)/cyclin B1 exclusively at mitosis (Figure 1). Notably, this process depends on the binding of PBK/TOPK to the CDK1/cyclin B1 complex at the mitotic spindle, which in turn induces its phosphorylation and thereby increases its binding ability to the CDK1/cyclin B1 complex through phosphorylation and inactivation of protein phosphatase 1 alpha (PP1α) [2,21,22]. These results all suggest that PBK/TOPK may be involved in the regulation of cellular proliferation and cell-cycle progression. 

In addition, the transcription of PBK/TOPK and its degradation varies with cell-cycle progression (Figure 1). The binding of the cell-cycle-specific transcription factors E2F and cyclic AMP-responsive element-binding protein/activating transcription factor (CREB/ATF) to the −146 and −312 bp binding sites within the PBK/TOPK promoter directly leads to its transcriptional upregulation [17,23]. C-Myc is functionally linked with E2F1 in controlling cell-cycle progression, and it activates PBK/TOPK transcription by indirectly enhancing E2F1 activity and cooperatively binding with E2F1 [24]. Checkpoint protein with FHA and RING domains (CHFR) is an E3 ubiquitin ligase that was demonstrated to ubiquitinate and mediate PBK/TOPK degradation, which is essential for its mitotic checkpoint function [25]. Src, a transforming protein with tyrosine kinase activity, was also identified as an upstream regulator of PBK/TOPK. It can directly bind and phosphorylate PBK/TOPK at Y74 and Y272, thereby enhancing its activity and enhancing PBK/TOPK stability, which allows it to avoid degradation after ubiquitination [26].

## 4. PBK/TOPK Function in Mitotic Progression and Tumor Cellular Proliferation 

**Figure 2:**
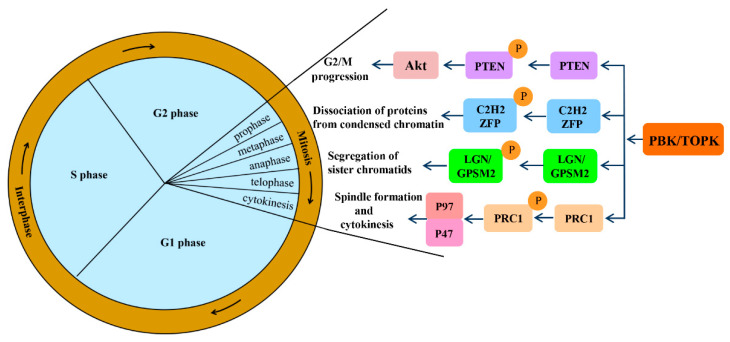
Signaling pathways involved in mitotic progression functions of PBK/TOPK. Abbreviations: PTEN, phosphatase and tensin homolog; Akt, also known as protein kinase B; ZFP, zinc finger proteins; LGN/GPSM2, Leu-Gly-Asn repeat-enriched protein/G-protein signaling modulator 2; PRC1, microtubule binding protein.

Mitotic progression is regulated by the co-ordination of several proteins and is crucial for the maintenance of genomic stability. PBK/TOPK is known to play pivotal roles in cellular mitosis; its main mitotic substrates and detailed functions in mitosis have been studied in depth (Figure 2). PBK/TOPK is of crucial importance in CHFR-mediated G2/M progression via phosphorylation and inactivation of PTEN, which results in activation of Akt [25]. Activated TOPK was also confirmed as the principal C2H2 zinc finger protein (ZFP) linker kinase that phosphorylates C2H2 ZFPs linkers within minutes in the prophase stage. This is a unique mechanism for reducing the DNA-binding activity of C2H2 ZFPs, which mediates the dissociation of hundreds of proteins from condensed chromatin during transition from prophase to telophase. PBK/TOPK was the first kinase to be identified as a master regulator of an entire family of transcription factors, based on their conserved motif [27]. At the late stage of mitosis, upregulated PBK/TOPK was shown to mediate the phosphorylation of LGN/GPSM2 (Leu-Gly-Asn repeat-enriched protein/G-protein signaling modulator 2) at Thr450, which might play a critical role in the segregation of sister chromatids and the formation of F-actin polymerization at the contractile ring [28]. In addition, activated PBK/TOPK was demonstrated to enhance the phosphorylation of the microtubule-binding protein PRC1 and ATPase protein p97 with p47 as an adaptor protein, which is indispensable for mitotic spindle formation and cytokinesis during mitosis [29]. 

**Figure 3:**
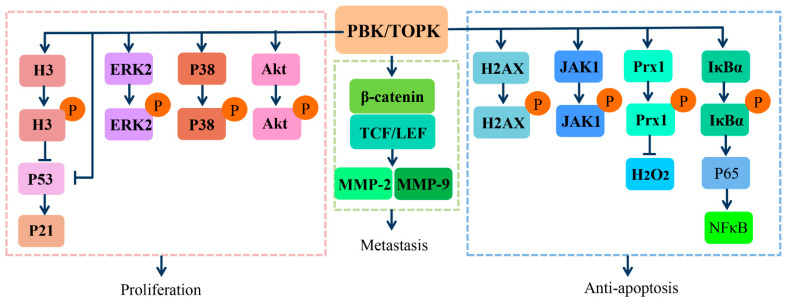
Signaling pathways involved in tumorigenic functions of PBK/TOPK. Abbreviations: H3, histone H3; ERK2, extracellular signal-regulated kinase 2; TCF/LEF, T-cell factor/lymphoid enhancer–binding factor; MMP-2/9, matrix metalloproteinase-2/9; JNK1, c-Jun-NH2-Kinase 1; Prx1, peroxiredoxin1; IκBα, inhibitor-κBα; NF-κB, nuclear factor kappa B.

PBK/TOPK overexpression in oncogenic pathways has been implicated in the proliferation, metastasis, and anti-apoptosis of tumors (Figure 3). Its overexpression allows the tumor cells to bypass the natural surveillance mechanism associated with the G2/M checkpoint and leads to aberrant entry into the mitotic phase by phosphorylating histone H3 at Ser10, as well as down-modulating the tumor suppressor p53, upregulating the cyclin-dependent kinase inhibitor p21, and thereby contributing to tumorigenesis [30,31,32]. Conversely, suppression of PBK/TOPK impaired its tumorigenic properties by reducing the phosphorylation and activation of mitogen-activated protein (MAP) kinase, including extracellular signal-regulated kinase (ERK) 2 and P38, inhibiting the expression of mutant p53, and inhibiting Akt activation [33,34,35,36,37,38]. Notably, phosphorylated ERK2 could in turn phosphorylate PBK/TOPK and increase its kinase activity, resulting in a positive feedback loop between PBK/TOPK and ERK2. PBK/TOPK also possesses the capacity to activate the interaction of transcriptional factor β-catenin, with its transcriptional coactivators T-cell factor/lymphoid enhancer-binding factor (TCF/LEF), which subsequently upregulates the transcription of matrix metalloproteinase MMP-2 and MMP-9, thereby facilitating the invasiveness and metastasis of tumor cells [16].

Aside from promoting proliferation and metastasis, activated PBK/TOPK could also alleviate As3+ treatment–induced apoptosis by binding with and phosphorylating histone H2AX at Ser139 in the nucleus. This may be responsible for the resistance of some melanoma cells to As3+ treatment [39]. Moreover, PBK/TOPK could inhibit UVB-induced apoptosis as well, by phosphorylating c-Jun-NH2-Kinase 1 (JNK1) at Thr183/Tyr185, activating it, and increasing the peroxidase activity of peroxiredoxin1 (Prx1) and decreasing the intracellular accumulation of H2O2 via phosphorylation Prx1 at Ser32 [40,41]. Moreover, TOPK directly phosphorylates inhibitor-κBα (IκBα) at Ser 32, which induces P65 nuclear translocation, nuclear factor-kappa B (NF-κB) activation, and subsequently leads to responsive transcription of anti-apoptotic genes [42,43,44]. These findings enrich our knowledge of the regulatory functions and underlying mechanisms of PBK/TOPK in mitotic progression of actively proliferating cells, especially tumors, and have led to a better understanding of its role in tumorigenesis. These studies all suggest that PBK/TOPK might serve as a diagnostic/prognostic indicator and therapeutic target in tumors. 

## 5. PBK/TOPK as Diagnostic/Prognostic Indicator and Therapeutic Targets in Tumors 

PBK/TOPK is upregulated in a wide range of mitotically active cancers; thus, a series of clinical studies have been conducted to investigate whether the levels of PBK/TOPK mRNA or protein have diagnostic or prognostic value [45]. In addition, the serine-threonine kinase function of PBK/TOPK drew the interest of many scientists and clinicians as a potential therapeutic target in highly proliferative malignancies. 

PBK/TOPK protein has been identified as a useful indicator for histopathological differentiation between cholangiocarcinoma and hepatocellular carcinoma, and low expression of PBK/TOPK is predictive of poor survival in cholangiocarcinoma patients [46]. PBK/TOPK overexpression could also predict poor prognosis, including shortened overall survival and time to recurrence, because of its effect on promoting tumor metastasis in patients with stage I lung adenocarcinoma and non-small-cell lung cancers (NSCLC) [36,37,47,48]. PBK/TOPK was also found to be significantly associated with the progression of human bladder cancer, nasopharyngeal carcinoma, and ovarian cancer [3,49,50]. In addition, overexpression of PBK/TOPK may lead to high malignant phenotype prostate cancer (PCa), while knockdown of PBK/TOPK suppressed cell proliferation, invasion, and migration of PCa cell lines in vitro [16,17]. In clinical settings, PBK/TOPK expression is an unfavorable prognostic indicator in patients with sporadic colorectal cancer with mutations in the proto-oncogene KRAS or BRAF and in patients with metastatic disease experiencing a response to anti-EGFR monoclonal antibody therapies [31,38]. Moreover, cytoplasmic PBK/TOPK was reported to be an independent prognostic marker for five-year survival of colorectal cancer patients, while higher expression of p-TOPK, but not TOPK, predicted poor prognosis, including significantly shorter progression-free survival (PFS) and overall survival (OS) in patients with primary central nervous system lymphoma [51,52]. Moreover, upregulation of PBK/TOPK in the peritumoral brain zone of glioblastoma multiforme (GBM) contributed to its vulnerability to tumor recurrence [53].

The inhibition of PBK/TOPK could benefit those colorectal cancer patients with a KRAS or BRAF mutation and those with metastasis, which represented 30–40% of all cases, and this may represent a new avenue of investigation for targeted therapy [54]. The upregulation of endogenous PBK/TOPK augmented resistance of human HeLa cervical cancer cells to TRAIL-induced apoptosis, while PBK/TOPK knockdown noticeably increased doxorubicin-mediated apoptosis [55]. This suggested that targeting PBK/TOPK may boost the efficacy of cervical cancer therapy with TRAIL or doxorubicin [42,43]. Suppression of PBK/TOPK not only impaired the proliferation of hepatocellular carcinoma cells but also resulted in an inhibitory effect of fibroblast growth factor (FGF21) treatment on D-galactose (D-gal)-induced hepatocyte apoptosis [34,35]. Although PBK/TOPK was upregulated in a variety of primary hematologic neoplasms, it was strongly downregulated in differentiated HL-60 cells after stimulation with phorbol ester (TPA) [56]. PKB/TOPK was also shown to mediate doxorubicin-induced growth arrest [23]. Moreover, reduction of PBK/TOPK expression decreased tumor growth and survival in high-grade malignant lymphomas, as well as in breast cancer cells [21,24,28,32]. Arsenite treatment was confirmed to cause phosphorylation of PBK/TOPK, which alleviated the apoptosis induced by arsenite treatment in melanomas. Thus, the combined inhibition of PBK/TOPK with arsenite treatment is a potential therapy for melanomas [39]. Moreover, PBK/TOPK activation following UVB irradiation also prevented apoptosis and induced skin cell transformation [40,41]. This suggests that PBK/TOPK is a potential target for the prevention and control of skin cancer, especially skin cancer induced by UVB.

The aforementioned studies highlight the potential of PBK/TOPK as a diagnostic and/or prognostic factor and a therapeutic target in different tumors. However, PBK/TOPK has also been shown to be overexpressed in normal proliferative cells, including sperm and neural precursor cells in the subventricular zone of the adult brain, and plays important roles in their physiological functions, including proliferation and self-renewal. Thus, further studies are warranted to further our understanding of its role under normal and pathological conditions before therapies targeting PBK/TOPK can be introduced into routine clinical practice. 

## 6. Involvement of PBK/TOPK in Myocardial, Renal and Cerebral Ischemia 

Ischemia is a pathological state that stimulates cellular proliferation. As PBK/TOPK is well-known to be upregulated in a variety of actively proliferating tissues, its specific roles in myocardial, renal, and cerebral ischemia have drawn our interest. 

PBK/TOPK has been shown to play instrumental roles in myocardial ischemia/reperfusion (I/R), ischemic preconditioning (IPC) and oxidative stress injury in H9C2 cardiomyocytes. PBK/TOPK inhibition aggravated H_2_O_2_ induced oxidative stress injury in cardiomyocytes, while overexpression relieved it by positively regulating the ERK pathway. Moreover, PBK/TOPK activation following IPC alleviated myocardial I/R injury. These results indicate that PBK/TOPK might mediate a novel survival signal in myocardial I/R and exert antioxidative stress effects by activating ERK signaling pathways [7]. The function of PBK/TOPK and the exact molecular mechanisms underlying renal protection rendered by remote limb ischemic postconditioning (RIPostC) have also been investigated. PBK/TOPK plays a vital role in RIPostC-mediated renoprotection. RIPostC could significantly protect kidneys against inflammatory cytokines and oxidative stress induced by ischemia/reperfusion injury, by increasing the phosphorylation of PBK/TOPK, PTEN, Akt, and GSK3β; nuclear translocation of Nrf2; and decreasing nuclear translocation of NF-κB. Inhibition of PBK/TOPK, using the inhibitor HI-TOPK-032, eliminated the renoprotective effects of RIPostC [8]. 

We have conducted a series of studies, to explore the function of PBK/TOPK in cerebral ischemic injury. We first reported the elevation of TOPK mRNA and its phosphorylated protein in the ipsilateral cortices of a focal cerebral ischemia/reperfusion model. Activated PBK/TOPK conferred neuroprotection against focal cerebral ischemia/reperfusion injury by its antioxidative effects, in part through activation of the extracellular signal-regulated kinase pathway [57]. Ischemic postconditioning (IPostC) protects against ischemic brain injury. We next explored the PBK/TOPK-related molecular mechanism in the antioxidant effect of IPostC against cerebral I/R. IPostC reversed the increase in oxidative stress within ischemic neural cells by activating PBK/TOPK, accompanied by increased levels of antioxidants Prx-1 and thioredoxin, as well as the activation of SOD. Akt pathway activation might initiate an irritative response to TOPK activation under IPostC treatment [6]. As the first line of defense against brain ischemia, microglia/macrophages respond dynamically to cerebral I/R injury [58,59]. We found that PBK/TOPK promoted microglia/macrophage polarization towards the M2 phenotype, by inhibiting HDAC1/HDAC2 activity, thus exerting neuroprotective effects against cerebral I/R [60]. These studies all suggest that PBK/TOPK is a potential target for the development of novel medicines to ameliorate myocardial, renal, and cerebral ischemia injury. Nevertheless, further studies are warranted to clarify the mechanism responsible for the neuroprotective effects of PBK/TOPK.

## 7. Research of PBK/TOPK Inhibitors

PBK/TOPK is highly and frequently activated in various actively proliferating cells, especially in cancers, and plays an indispensable role in tumor development, metastasis, chemoresistance, and ischemia. Due to the identification of PBK/TOPK as a promising novel therapeutic target, inhibitors of PBK/TOPK have been developed. Interestingly, even coffee and its active ingredient, caffeic acid (CaA), were demonstrated to reduce the risk of cancers, by suppressing PBK/TOPK activity [61]. Several potent PBK/TOPK inhibitors have been developed, including HI-TOPK-032 and OTS514/OTS964, among others, and they are described in detail below.

In 2012, using sequence homology of TOPK and MEKs, Kim et al. screened 36 drug candidates with similar structures to MEK inhibitors as possible PBK/TOPK inhibitors. Using an in vitro PBK/TOPK kinase assay and a cell proliferation assay, they identified HI-TOPK-032 as a potent PBK/TOPK inhibitor. Moreover, HI-TOPK-032 was confirmed to inhibit PBK/TOPK kinase activity without affecting the activity of other MAP kinase family members, such as ERK1, JUK1, or P38. Computer modeling indicated that HI-TOPK-032 bound to the PBK/TOPK active site [62]. Subsequent studies on its efficacy and therapeutic potential against colorectal cancers and other human cancers have been conducted. HI-TOPK-032 was shown to inhibit colon cancer cell growth and increase apoptosis, by reducing ERK-RSK phosphorylation, as well as regulating the expression of p53 [62]. In addition, treatment of the glioma initiating cell (GIC) population in glioblastomas with HITOPK-032 almost completely abolished the growth of experimentally induced subcutaneous GBM tumors in mice, without any side effects or toxicity [63]. Adult T-cell leukemia/lymphoma (ATLL) is an aggressive neoplasm of CD4+ T cells linked to human T-cell leukemia virus type 1 (HTLV-1) infection. HI-TOPK-032 was found to decrease the proliferation and viability of both HTLV-1 transformed T-cell lines and ATLL-derived T-cell lines in a dose-dependent manner. This was consistent with the results of ATLL xenograft models [4]. These studies suggest that HI-TOPK-032 is a promising therapeutic target for clinical use in tumor treatment. 

Using high-throughput compound library screening and extensive structure–activity relationship (SAR) studies, Matsuo et al. discovered that some thieno[2,3-c]quinolone compounds possessed the ability to inhibit TOPK kinase. Among them, OTS514 {(R)-9-(4-(1-aminopropan-2-yl)phenyl)-8-hydroxy-6-methylthieno[2,3-c]quinolin-4(5H)-one)} was identified as an extremely potent PBK/TOPK inhibitor, with a median inhibitory concentration (IC_50_) value of 2.6 nM. Moreover, they developed an OTS514-analog compound, OTS964, through extensive SAR studies. Although OTS964 was less effective at inhibiting PBK/TOPK activity than OTS514, with an IC_50_ value of 28 nM, this dimethylated derivative compound had higher bioavailability. Both compounds induced complete lung tumor regression in murine xenograft models, by causing a cytokinesis defect and subsequent cellular apoptosis, when delivered either orally or intravenously [64]. Moreover, both compounds abolished the ex vivo growth of patient-derived ovarian cancer cells in a dose-dependent manner and completely suppressed dissemination of ovarian malignancy in peritoneal xenografts [3]. However, free OTS964 and OTS514 triggered severe weight loss and adverse hematopoietic toxicity, when administered to mouse xenograft models. To overcome this problem, OTS964 was encapsulated into liposomes, to improve tumor-specific uptake through the enhanced permeation and retention effect. The liposomal delivery of OTS964 prevented anemia and leukocytopenia. Moreover, it enabled full recovery following oral administration and caused tumor shrinkage even after discontinuation of treatment, which was not observed with OTS514. The liposomal formulation of OTS964 is now a leading candidate for clinical development [64,65]. More recently, the labeling of a PBK/TOPK inhibitor with F-18 has been exploited for positron emission tomography (PET) imaging, which is used clinically for accurate identification of patients, their stratification, and disease monitoring. Furthermore, [18F]- labeled OTS964 showed favorable pharmacokinetics and biodistribution when injected intravenously in a glioblastoma-carrying mouse model. Moreover, [18F]FE-OTS964 was the first PBK/TOPK inhibitor to be used for imaging purposes and proved useful in the continued investigation of the pharmacology of PBK/TOPK inhibitors and the biology of PBK/TOPK in cancer patients [66]. 

Apart from the potent selective PBK/TOPK inhibitors mentioned above, several other inhibitors have also been studied. The compound ADA-07 (5-((1s, 3s)-adamantan-1-yl)-3-(hydroxyimino) indolin-2-one) was also identified as a novel PBK/TOPK inhibitor. Homology modeling and subsequent molecular docking simulated the interaction of ADA-07 with PBK/TOPK at the ATP-binding pocket. Subsequently, pull-down assay, ATP competition, and in vitro kinase activity assays were conducted to confirm the binding and inhibitory effect of ADA-07 on PBK/TOPK [67]. Topical application of ADA-07 effectively inhibited SUV-induced skin carcinogenesis, by suppressing activation of MAPK signals in mouse skin, following exposure to solar irradiation [68]. MicroRNA (miR)-216b, as a tumor suppressor, was downregulated in various cancer types. Moreover, miR-216b-3p was found to inhibit lung adenocarcinoma cell growth by directly binding PBK/TOPK and negatively regulating its expression. Therefore, PBK/TOPK may also be considered as a therapeutic target with microRNA-mediated regulatory control [69]. A series of 1-phenyl phenanthridin-6 (5H)-one derivatives have also been identified as a novel chemical class of PBK/TOPK inhibitors, among which **9g** compound displayed superior anticancer activity, even compared to OTS964, against colorectal cancer [70]. Glycyrol (GC), a representative coumarin compound isolated from licorice, was able to bind strongly to PBK/TOPK and inhibited its kinase activity. It not only displayed a highly inhibitive effect against several human NSCLC cell lines, but also significantly suppressed tumor growth in vivo [71]. SKLB-C05 was developed as a PBK/TOPK selective inhibitor with sub-nanomolar inhibitory potency. Oral administration of SKLB-C05 at concentrations of 20 and 10 mg/kg/d dramatically attenuated colorectal carcinoma (CRC) tumor xenograft growth and completely suppressed hepatic metastasis of HCT116 cells. This antitumor and anti-metastatic activity made SKLB-C05 a potential PBK/TOPK inhibitor that may be useful in treating a variety of malignancies [72]. 

PBK/TOPK is becoming an attractive target in chemotherapeutic drug design because of its important role in cancer progression. In silico structural and functional analyses of PBK/TOPK, by structure modeling and molecular dynamics studies, not only identified the pathomechanisms of PBK/TOPK inhibitors, but also provided a novel method for the structure-based drug design of inhibitors. Based on the development of new technologies and methods, more specific PBK/TOPK inhibitors are expected to be discovered [73,74]. However, further in vitro and in vivo studies are needed before PBK/TOPK can be considered for clinical application.

## 8. Conclusions

Due to its distribution, protein kinase activity, and specific roles in the mitotic progression and cellular proliferation of actively proliferative cells, PBK/TOPK has drawn the attention of many scientists and clinicians seeking to understand its involvement in tumor proliferation, metastasis, and chemoresistance and to elucidate its potential as a diagnostic and prognostic marker, as well as a therapeutic target in various types of cancers. Specific inhibitors of PBK/TOPK have been discovered. However, as more research has been conducted to evaluate of the function of PBK/TOPK in mitotically active cells other than tumors, more biological functions and roles during pathological progression have been discovered. In addition to its roles in tumors, PBK/TOPK has been found to be distributed in normal tissues, playing essential roles during physiological processes, such as spermatogenesis and neuronal self-renewal. Moreover, studies have revealed a role of PBK/TOPK in ischemic injury, including cardiac, renal, and cerebral ischemia, and have demonstrated that it mediates the protective effects of ischemic postconditioning against ischemia. The latest study also presents evidence that PBK/TOPK plays a supporting role in checkpoint kinase 1 (CHK1)-mediated maintenance of DNA replication fidelity [75]. Therefore, it is crucial that further research is conducted to elucidate the essential functions of PBK/TOPK before we can consider its applicability in antitumor therapy.

## Data Availability

No new data were created or analyzed in this study. Data sharing is not applicable to this article.
